# Descriptive study of adolescents hospitalized in the Psychiatric Unit of a hospital in Madrid, Spain

**DOI:** 10.1192/j.eurpsy.2024.249

**Published:** 2024-08-27

**Authors:** P. Del Sol Calderon, A. Izquierdo de la Puente, R. Fernández, M. García Moreno, L. Mallol, I. Palanca

**Affiliations:** ^1^Psychiatry, Hospital Puerta de Hierro; ^2^Psychiatry, Hospital Universitario Infanta Cristina, Madrid, Spain

## Abstract

**Introduction:**

An increase in suicidal behavior among the adolescent population is reflected in the literature and in clinical practice. According to a study of suicidal behavior and mental health by the Spanish ANAR Foundation, the number of cases with suicidal behavior has experienced an accentuated growth in the period 2012-2022 (1,921.3%), highlighting the increase produced in the post-COVID-19 period, between 2020 and 2022 (128%)

**Objectives:**

To analyze the reasons for admission to the short hospitalization unit. To describe the sociodemographic characteristics of hospitalized adolescents.

**Methods:**

Descriptive observational study of the sample of adolescents admitted to the inpatient psychiatric unit of the Hospital Universitario Puerta de Hierro between January 1, 2023 and June 30, 2023. It is carried out through the information obtained in the clinical history of the patients.

**Results:**

During this period of time 113 adolescents were admitted, 80.2% were female. The mean age was 15.16 years. The main reason for admission was autolytic ideation, occurring in 33.3% of the patients. The second most frequent reason for admission was suicide attempt (29.7%) and behavioral disturbance (17.1%) was the third most frequent. Of the methods used in suicide attempts, drug overeating stands out among the methods used in suicide attempts. (75.8%), followed by attempted hanging (12.1%) or cutting (12.1%).

**Image:**

**Figure:**
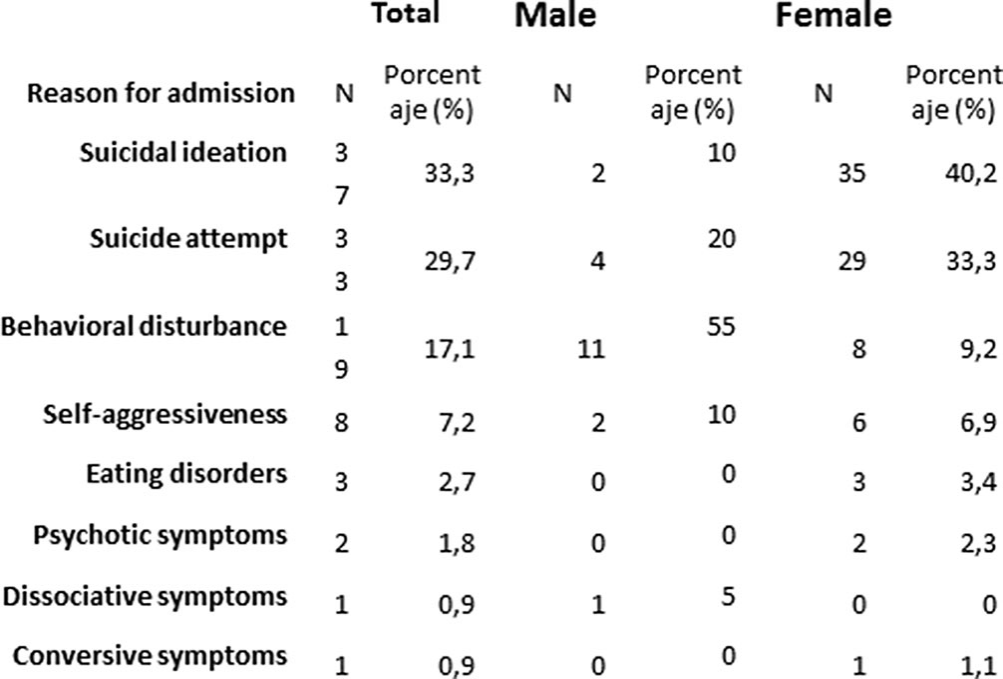

**Conclusions:**

The results corroborate what is reported in the scientific literature, where self-harm and self-injury attempts have increased and are the most frequent reasons for admission. This shows that suicide is a public health problem of the first order, where prevention and early intervention programs are necessary.

**Disclosure of Interest:**

None Declared

